# AVASCULAR NECROSIS: A RARE COMPLICATION OF STEROID THERAPY FOR PEMPHIGUS

**DOI:** 10.4103/0019-5154.39739

**Published:** 2008

**Authors:** Vibhu Mendiratta, Anamita Khan, R S Solanki

**Affiliations:** *From Department of Dermatology and Venereology and Radio Diagnosis, Lady Hardinge Medical College and Associated Hospitals, New Delhi - 110 001, India*

**Keywords:** *Avascular necrosis*, *femur*, *pemphigus*

## Abstract

A patient of pemphigus vulgaris presented with avascular necrosis of the femur after long-term high-dose corticosteroid therapy. Corticosteroids used on a long-term basis can cause avascular necrosis of bone and this has been seen in various diseases. This is attributable to both the disease process itself and the therapy i.e. corticosteroid usage. In dermatological practice avascular necrosis of bone has been seen more commonly with SLE and also with psoriasis using long-term steroids. Avascular necrosis in a case of pemphigus on steroid therapy is a rare finding. We report such a case of pemphigus vulgaris developing avascular necrosis of bone following corticosteroid therapy.

## Introduction

Corticosteroid therapy, irrespective of the mode of administration, systemic, oral or parenteral or topical therapy has its own attendant risks of corticosteroid-induced side-effects with a greater associated risk of developing the same with systemic therapy. Avascular necrosis of the femur head is one of the universally recognized side-effects of steroid therapy. The etiology of avascular necrosis is multifactorial and ranges from traumatic namely fracture of the neck of femur,^1^,^2^ traumatic dislocation of hip,^4^ to slipped femoral capital epiphysis.^5^ It has been reported to occur secondary to osteotomy,^6^ sickle cell anemia,^7^ alcoholism,^8^ autoimmune connective tissue disorder like rheumatoid arthritis, lupus erythematosus, pancreatitis, occlusive vascular disease, pheochromocytoma, infections, radiation, Gaucher's disease, renal transplantation, Cassion's disease and even in pregnancy.^6^

Amongst the dermatological diseases, avascular necrosis of the femoral head has been reported more commonly in SLE,^7^ psoriasis,^8^ secondary to even topical therapy to potent corticosteroids such as clobetasol cream,^9^ in patients on steroid therapy for pemphigus,^10^ sarcoid, eczema^10^ etc.

Avascular necrosis is clinically characterized by gradual onset of pain in motion, relieved by rest in the affected joint with radiation down the affected limb at times leading to muscle spasms. A slight limp, often unilateral with limitation of motion to a variable degree is characteristic. The patient may have an abductor lurch and rotation, with limitation of abduction and adduction. Atrophy of the proximal muscles may be associated. Head of the femur has minimum blood supply with few anastomoses leading to a wedge-shaped area of avascular necrosis. Histologically the involved bone has three zones - necrotic, granulomatous and a variable zone. Radiologically the picture is variable depending on the stage of the disease but a wedge-shaped area of increased radio-opacity with the base adjacent to the articular cartilage and the apex pointing to head of the involved bone. Necrosis appears as a mottled area and the fibrous zone as a radiolucent band with demineralization of the uninvolved bone.^11^

Various mechanisms have been put forth in respect of the etiopathogenesis of this crippling side-effect, namely increase in the intra-osseous pressure resulting from lipocyte hypertrophy and derangements in fatty metabolism causing deposition of fat in the marrow spaces of the skeleton in patients who were treated with steroids,^12^,^13^ particularly in individuals who underwent short-term treatment with high-dose steroids.

In addition in autoimmune diseases a correlation has been seen between glucocorticoid and bone death, which appears to be due to blood stasis and ischemia in the trabecular bone. Formation of thrombosis or fat embolism has proven invalid.^14^ Increases in the osteocyte apoptosis owing to micro damage in the bone^15^ could have a role too.

Whatever may be the pathologic etiology for the avascular necrosis, so far an exact relationship between the dose and the mode of administration of steroid and the risk of developing avascular necrosis has not been determined.

Reports of avascular necrosis in patients of pemphigus vulgaris on steroids are rare. We report avascular necrosis of bilateral hip (head of femur) in a young Indian patient of pemphigus vulgaris.

## Case History

A 25-year-old unmarried male presented with peeling of the skin all over the body after formation of flaccid bullae and oral erosions of recent onset. The patient complained of burning sensation on eating food and oral erosions of one month duration after which he started developing flaccid bullae that ruptured in a period of one to three days to leave behind erosions. On examination, flaccid bullae were found on the limbs with positive Nicolsky's sign and bulla spread sign. Oral mucosa showed erosions. Genital mucous membrane was spared.

**Fig. 1:**
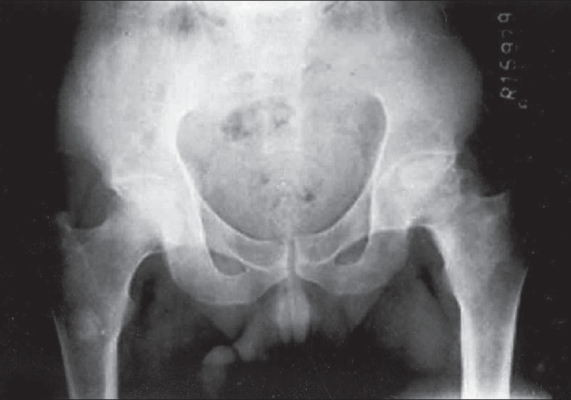
X- ray hip joint shows area of lucency and sclerosis on the proximal femur

Histopathology of the skin tissue showed suprabasal split and the patient was diagnosed as a case of pemphigus vulgaris. The patient was started on 3 ml dexamethasone I/V (equivalent to 64 mg prednisolone) to control the disease and tapered to 2 ml (43 mg prednisolone) in seven days interval. The latter was continued for 20 days as new lesions kept on erupting. The patient was tapered to 40 mg prednisolone that was maintained for a period of four months. The dose was tapered to 20 mg prednisolone for another two months and steroid was stopped as the lesions (both cutaneous and oral) healed completely. The disease recurred in one and a half years in a milder form and the patient was started on 2 ml dexamethasone (43 mg prednisolone) for a period of 10 days followed by 40 mg prednisolone. The cutaneous lesions healed with healing of few mucosal lesions. The patient was also started on methotrexate 7.5 mg/week, increased to 15 mg/week. The patient stopped methotrexate on his own. The disease recurred after a period of seven months and the patient was started on 60 mg prednisolone, tapered to 50 mg after a period of 10 days and continued on 40 mg for a month. The patient also contacted pulmonary tuberculosis. He was prescribed anti-tuberculosis for a period of one year. Rifampicin aggravated the disease so it was stopped and patient was given I/M streptomycin for two months of intensive phase. The disease recurred for the fourth time after nine months with a few cutaneous lesions and no oral lesions. The patient also complained of pain in hip joint on movement. The patient was started on 40 mg prednisolone a day. The cumulative dose of corticosteroids taken by the patient in the last four years was approximately 11 g. The patient was concurrently administered calcium tablets all through his disease. X-ray hip joint revealed mottling of the trabecular bone and area of lucency and sclerosis on the proximal femur (Fig. 1). Bone scan showed avascular necrosis right femoral head, sacroilleitis, stress fracture of the infra-trochanteric area of the left femur and reduced vascular flow in the left femoral head inconsistent with his age. Orthopedic evaluation described it as avascular necrosis and he was advised bed rest, non-steroidal anti-inflammatory drugs, tapering of steroids and total hip replacement.

## Discussion

Amongst the non-traumatic causes of avascular necrosis of the femur, patients of SLE who are treated with corticosteroids have been reported to have a high relative risk of developing avascular necrosis with a reported frequency of 5-40%.^16^

The first patient of pemphigus (described by Pietrogrande and Mastronmarino) who developed hip destruction due to corticosteroid therapy was a woman who developed avascular necrosis after four years of therapy with 100 mg cortisone acetate daily followed by a report of three cases with pemphigus developing avascular necrosis of femur.^17^

The duration of steroid treatment, the total cumulative dose and the highest daily dose of steroids have been implicated as important factors in the development of avascular necrosis. The incidence of males with osteoporosis is less as compared to females whether the patient is on long-term steroid or otherwise.

One study in SLE patients reviewed the clinical features of the disease and the laboratory data of the patients of SLE at the time of developing avascular necrosis and the dose of steroids and found that a rash introduction of high-dose steroids (>30 g/d) prednisolone without “steroid preloading” was associated with a higher risk of avascular necrosis and suggested that a minimum/ moderate amount of glucocorticoid preloading lessens the risk of avascular necrosis.^18^

Corticosteroids even in high doses do not cause avascular necrosis of the femoral head in the majority of patients. It could be that certain risk factors pertaining to the particular disease entity might enhance the risk e.g. hyperuricemia in psoriatics, hyperlipidemias in SLE etc. At the same time some behavioral addictive problems like alcoholism might act as a contributory factor to hasten the osteonecrosis in case of corticosteroid administration in such patients. Steroid-induced osteoporosis should also be monitored and timely management of the same needs to be instituted in order to prevent avascular necrosis.

Review of the literature of avascular necrosis in pemphigus gave insufficient data on the actual prevalence of avascular necrosis in pemphigus patients and the disease-specific risk factors for developing the same. Since pemphigus patients need long-term administration of high-dose steroids they are at a higher risk of developing avascular necrosis (symptomatic/asymptomatic); we would therefore like to recommend pretreatment bone densitometry of the lumbar spine in patients of pemphigus having a severe disease, management of complicating factors like osteoporosis and alcoholism and a baseline MRI of the femoral head and at six months and at 12 months interval thereafter in order to screen the patients for any asymptomatic avascular necrosis and its early treatment.
